# Income and Career Concerns Among Emerging Adults From Finland, Sweden, and the United Kingdom During COVID-19

**DOI:** 10.1177/21676968231153691

**Published:** 2023-01-20

**Authors:** Julia A. Nuckols, Gintautas Silinskas, Mette Ranta, Terhi-Anna Wilska

**Affiliations:** 1 Department of Social Sciences and Philosophy, 4168 University of Jyväskylä, Jyväskylä, Finland; 2 Department of Psychology, University of Jyväskylä, Jyväskylä, Finland; 3 Department of Education, University of Helsinki, Helsinki, Finland

**Keywords:** COVID-19, emerging adults, young people, income concerns, career concerns

## Abstract

In this study, we examine the prevalence of income and career concerns among emerging adults in three different welfare states during COVID-19: Finland (*n* = 309), Sweden (*n* = 324), and the United Kingdom (*n* = 343). This study also delves into how factors such as one’s self-perceived financial situation, generalized mistrust, loneliness and socio-demographics are related to emerging adults’ income and career concerns. Results showed that individuals from the United Kingdom were more likely to experience increased income and career concerns than those in Finland and Sweden. Our results also suggest that income concerns were associated with one’s current financial situation, future financial situation, childhood financial situation, and loneliness. Also, career concerns were related to generalized mistrust, loneliness, and age. For both country-specific and general analyses, loneliness emerged as the most important for increased income and career concerns for emerging adults in all three countries.

## Introduction

The COVID-19 pandemic has been impactful to the lives of many, with studies reporting increased distress among emerging adults in particular (Halliburton et al., 2021; Preetz et al., 2021; Ranta et al., 2020; Wilska et al., 2021). Disruptions in everyday life, social lives, work, and education, as well as increased loneliness and insecurity have implications on the development of emerging adults (Halliburton et al., 2021; Wilska et al., 2021). While emerging adulthood is a phase characterized by uncertainty and increased demand in social support, facing sudden obstacles that exceed emerging adults’ capacities may lead to increased stress and possible maladaptive behaviors, as seen in previous crisis situations such as the 2008 financial crisis (Grob et al., 2001; Ranta, 2015). Therefore examining certain COVID-19 pandemic-related aspects, such as emerging adults’ current and future financial situation estimates, generalized mistrust and loneliness, and how they relate to income and career concerns amongst emerging adults elicited research interest. Studying how these factors are linked during the pandemic among emerging adults will identify how policymakers and social structures can better support emerging adults’ experiences of heightened stress over their finances and careers.

This study focuses on the income and career concerns of emerging adults aged 18–34 in Finland, Sweden, and the United Kingdom during the COVID-19 pandemic. The different pandemic strategies and pandemic related outcomes in each country, as well as differences in welfare state models raised our interest for comparative research. Finland and Sweden are both Nordic welfare states and considered culturally similar, while the United Kingdom belongs to the Liberal welfare state regime (Esping-Andersen, 1990). Nordic welfare states are built upon universal and social insurance systems with an emphasis on maximized labor force participation, equality, and income redistribution (SSA, 2018; Esping-Andersen, 1990). Liberal welfare regimes, such as the United Kingdom, are built upon a social insurance and social assistance system, however an emphasis is put on minimal government interference and the free market economy (SSA, 2018; Esping-Andersen, 1990). On a global scale, all three countries score relatively high on the quality-of-life index (Numbeo, 2019) and have fairly low poverty rates. Finland and Sweden, however, do differ from other European countries as both countries rank lower than the European average in poverty rates (Office for National Statistics, 2017). According to statistics, the poverty rates for all three countries in 2019 were Finland (6.5%), Sweden (8.9%), and the United Kingdom (11.7%) (Statista, 2019). However, poverty in these three countries can look very different. For instance, homelessness in Finland is measured at approximately 9 persons per 10,000 inhabitants, 32 per 10,000 in Sweden, and 54 per 10,000 in the United Kingdom (The Housing Finance and Development Centre of Finland, 2021; SVT, 2019; Shelter, 2019).

The United Kingdom implemented the strictest preventative measures out of the three countries (i.e., full lockdowns, cautious reopening of society, low-threshold intervention). Finland took some legally enforceable measures to control the pandemic, such as limiting recreational activities and food and drink services, while issuing strong recommendations to mitigate the spread of the virus. Sweden had a lenient approach, with much of the pandemic being controlled by recommendation-based actions and a strong reliance on personal responsibility. Sweden also eventually implemented legally enforceable measures but remained the most lenient out of the three countries studied (Wilska et al., 2021).

The sudden shifts in societal functions and one’s everyday life have been a catalyst for unpredictability when industries have closed and reopened or been under strain as both society and individuals aimed to limit viral exposure (Wilska, et al., 2021). Widespread remote work and study left populations suddenly isolated from normal social functions, leading to increased loneliness (Lee et al., 2020). The strain on young people has been particularly noticeable as many young individuals are either students or at an age typical for workers in the affected industries (travel, entertainment, hospitality). Young people often have less-established careers and financial security, making them particularly vulnerable to the sudden changes induced by, for instance, the pandemic (Ranta, 2015). Individuals and families have also lost or been at risk for losing loved ones due to the disease, adding the need for authorities to make the right decisions that protect the individual from both the health and financial implications of the pandemic. These effects call upon both authoritative and individual responsibility, thus adding an element of trust to the effects of the pandemic. This elicits interest in how issues regarding one’s income and/or career may have different consequences in different countries and how much emphasis is put on support from family and friends in the event of such issues.

The current study analyzes international survey data collected in April 2021, approximately a year into the COVID-19 crisis from the three countries (Finland, Sweden, and the United Kingdom). These data cover several topics regarding the pandemic, from disruptions in everyday life to changes in lifestyle, to concerns and opinions on the disease itself and the effects on society caused by both the virus and government intervention. We compared the prevalence of emerging adults’ income and career concerns in all three countries, as well as how one’s self-perceived financial situation (i.e., current, future, and childhood financial situation), generalized mistrust, loneliness, and socio-demographics (i.e., highest education, parental education, age, and gender) differentially relate to the aforementioned concerns. We approach the research objectives from the perspective of life course theory in the context of societal crises in different welfare states.

### Employment and Financial Uncertainty during COVID-19

Emerging adulthood is a stage characterized by important decision making and pathway establishment for one’s adult life. It is also marked by uncertainty, possibilities, and adaptation to external and internal social and economic conditions (Arnett, 2014; Elder & Rockwell, 1979). While the past few decades have led to more freedoms for emerging adults on how they carry out their developmental tasks, these freedoms have also introduced rising precarity and uncertainty (Silva, 2012). The timing of these developmental tasks has also become more prolonged, with increased job experimentation and relocation. (Lanz et al., 2021). Additionally risks involving social and financial exclusion may lead to NEET (not in education, employment, or training) circumstances, to which emerging adults are particularly vulnerable to during critical life stage transitions (Bynner & Parsons, 2002). The isolating nature of the pandemic, as well as its impact on financial and career issues have the potential to increase said vulnerability among emerging adults.

The COVID-19 pandemic has caused challenges for emerging adults with its financial impact being the most prominent (Ranta et al., 2020). The societal consequences of lockdowns, disruptions in work and studies, limited mobility and precarious employment all have potential implications for emerging adults. Unexpected global events have the potential to directly impact expected transitions (e.g., graduation, finding work and establishing a career, purchasing a home), which can lead to decreased well-being and life satisfaction (Hendry & Kloep, 2007b), anxiety (Hendry & Kloep, 2007a), maladaptive behaviors, and future precarity (Côté & Bynner, 2008). The developmental tasks of obtaining a degree, establishing a career, achieving financial independence, and building one’s professional social connections are all considered important in the life stage of emerging adulthood, with finding employment being particularly important in terms of well-being and life satisfaction (Furstenberg et al., 2005; Recksiedler & Landberg, 2021; Scharf et al., 2004; van Lill & Bakker, 2020).

Settersten et al. (2020) have postulated that the implications of the pandemic on current emerging adult cohorts may lead to precarious employment, unemployment, increased loneliness and compromised lifelong earnings. This, coupled with the initial economic shock of the pandemic in an already precarious labor market, may lead to amplified concerns amongst students, graduates and jobseekers or precarious workers (Settersten et al., 2020). From a life course perspective, it is also worth considering how short-term unemployment, such as those witnessed during the pandemic, translate into long-term unemployment and precarity, particularly amongst young, less-experienced individuals in the job market (Settersten et al., 2020). As Salmela-Aro et al. (2015) have found, succeeding in these important developmental tasks increase well-being, and therefore the consequences of increased stress, concern, and potentially compromised circumstances to complete these tasks may be harmful to the development of emerging adults.

The COVID-19 pandemic has led to closures and restrictions of certain industries. While the initial fears involving economic recession have passed since the beginning of the pandemic, many industries still struggle with uncertainty of continuity, particularly those in the culture and hospitality sector (Wilska et al., 2021). Previous economic crises have already evidenced that the consequences extend beyond the loss of income with unemployment, leading to perturbed well-being and mental health issues (Preetz et al., 2021). Previous research on the current pandemic indicates that it has an adverse effect on the economic situations of emerging adults with the loss of internships, jobs, and limited job offers (Aucejo et al., 2020; Ranta et al., 2020). These conditions may also lead to prolonged insecurity and increased worry about financial security and future prospects, causing higher stress and anxiety during an already uncertain life stage (Elmer et al., 2020).

### COVID-19, Loneliness and Trust

The COVID-19 pandemic has had detrimental effects on continuity and lifestyle regularity (Glowacz & Schmits, 2020). Studies have indicated increased loneliness during the COVID-19 pandemic due to social distancing and isolation guidelines, which in turn has added mental strain (Lee et al., 2020; Wilska et al., 2021). Remote work and education have also challenged the availability of regular social contact for many individuals (Wilska et al., 2021). While struggling with mental distress caused by the pandemic as well as the developmental tasks of emerging adulthood in general, limited or stifled social support have the potential to further amplify mental health problems and anxieties (van den Berg et al., 2021). In addition to remote work, those who have lost jobs are also susceptible to increased loneliness due to the pandemic, as studies have shown that unemployment is related to increased loneliness (Morrish & Medina-Lara, 2021). Additionally, peers and friends are believed to be one of the most important sources of social support for emerging adults. Since the formation of serious romantic relationships is considered an important developmental task of emerging adulthood (Arnett, 2000; van den Berg et al., 2021), those lacking intimate relationships may also be susceptible to increased loneliness particularly during the pandemic, adding an additional factor in how the pandemic increases inequalities. Studies from the United Kingdom showed that loneliness during lockdowns was particularly high in areas with young populations, high unemployment and prevalent among individuals who were young and single (Office for National Statistics, 2021). Therefore, loneliness in terms of income and careers is worth exploring, as increased loneliness has been a key element in COVID-19 research. Furthermore, it gives way to how loneliness relates to income and career concerns as it may be telling of less available peer/family support, as well as loss of work or, for instance, concerns regarding job performance after shifting to remote working conditions.

The COVID-19 pandemic is a societal crisis in which experts and authorities need to mitigate the risk and potential harms to the public, thus leading to a situation where individuals are vulnerable to the decision making of institutions and the actions of others outside their immediate agency. For the sake of this study, generalized trust was measured to investigate how generally trustworthy other individuals are (Mayer et al., 1995). Generalized trust has been positively attributed to compliance and negatively with perceived risks, making it a potential tool to understand how individuals perceive COVID-19 as a risk and how likely they are to comply with the recommendations and rules of public and health authorities (Ahorsu et al., 2022). With generalized trust being found to be negatively correlated with the perception of risks (Siegrist et al., 2005), therein lies the possibility that these effects expand into the perceptions of one’s financial security and sustainability. Generalized trust can also be utilized in examining impressions and satisfaction in political entities (Nannestad, 2008). With civic and political identities largely being formed in emerging adulthood (Walker & Iverson, 2016), the politicized nature of the pandemic makes generalized trust an interesting angle to consider.

### Welfare States in Support of Life Course Development

Given that this is a cross-national study focusing on a global crisis, it is important to postulate upon the effects of one’s sociocultural environments and societal constructs on emerging adulthood development. In Esping-Andersen’s (1990) theory on *welfare capitalism*, there is a suggested threefold typology of social democratic, conservative, and liberal welfare state regimes. Previous life course research has shown the importance of political, economic, and cultural characteristics when researching emerging adults’ life course development from a comparative perspective (Bertogg, 2020). Therefore, life course research may incorporate the work of Esping-Andersen when examining generalized sociopolitical and economic influences in life course development, as seen in the works of Buchmann and Kriesi (2011), Möhring (2016) and Stier et al. (2001). According to Esping-Andersen (1990), the Nordic countries offer citizens services and benefits to better social security, insofar that citizens may sustain their livelihood regardless of their labor market status. In the same welfare structure, students, for example, are provided benefits in the form of student allowances and scholarships, with some minor country-specific differences (Social Insurance Institution of Finland, 2022; The Swedish Board of Student Finance, 2021). Both countries also provide support in the form of unemployment benefits, universal income benefits, as well as universal health care.

Reports have shown that the amount of government spending on unemployment subsidies grew in Finland during the pandemic due to high rates of furloughs particularly in the most affected fields as furlough programs were made more generous (Juranek et al., 2020). Similarly, Sweden introduced its own furlough program for pandemic related loss of work, with furloughed employees receiving 75–80% of their lost salary. Both Finland and Sweden underwent shocks in their labor markets, however the effects were less notable in Sweden, likely due to the leniency of their measures (Juranek et al., 2020).

In the United Kingdom, which is classified as a liberal welfare regime (Esping-Andersen, 1990; Powell & Barrientos, 2004), welfare systems are more privatized. This elicits more market freedoms and less regulation in employment and businesses in comparison to social democratic regimes such as Finland and Sweden (Esping-Andersen, 1990; Meyer et al., 2009). Liberal regimes such as the United Kingdom emphasize the importance of self-reliance and early labor market entry, while providing limited means of state provided safety nets (Arundel & Ronald, 2016). Some forms of social support are present in the United Kingdom; however, they are less accessible than in Finland or Sweden, with usually more caveats in order to be eligible for them (GOV.UK, 2022).

The United Kingdom has faced economic challenges in two regards, one involving the pandemic and the other involving Brexit (De Lyon & Dhingra, 2021). In the early months of the pandemic, the gross domestic product of the United Kingdom fell by around 20% before partially recovering over the summer, however the continued state of the pandemic and new lockdowns further affected the economy (De Lyon & Dhingra, 2021). As the United Kingdom left the European Union in January 2021, a large loss in volume of goods trade was stark. The United Kingdom did, however, implement interventions that would lessen the impact of these two events, including the extensive Job Retention Scheme (JRS) as well as other business support measures which aimed to ease the economic activity in returning to its usual levels after restrictions were lifted. Nevertheless, some still predict that unemployment will continue to be higher than prior to the pandemic up until 2024 (De Lyon & Dhingra, 2021). What is notable is that unlike Finland and Sweden, the United Kingdom focused its governmental assistance on businesses and institutions, not so much on individuals, while some programs were set into place to better the resilience of civilians impacted by the pandemic.

Settersten et al. (2020) forecast that the levels of uncertainty elicited by the pandemic, both long and short term, are with high probability linked to one’s age, social class, gender, race/ethnicity, and the welfare system of the country individuals live in. The disruptions in one’s life course development in countries with little institutional support therefore demand more assistance from networks of family and friends, as well as former employers and college career centers, for example (Settersten et al., 2020). However, when these disruptions happen en masse, therein lies a question of resource adequacy for both liberal and social democratic regimes. From a social policy point of view, it is worth discussing how the availability of social security supports can affect and potentially amplify concerns regarding one’s career and financial concerns (Chung et al., 2012).

### The Present Study

This study aims to examine the income and career concerns of emerging adults from Finland, Sweden, and the United Kingdom during the COVID-19 pandemic, and to investigate their associations with self-perceived financial situation (current, future, and childhood), generalized mistrust, loneliness as well as sociodemographic variables (highest education, parental education, age, gender), and country. The research questions (RQs) examined are:

RQ1: To what extent do emerging adults’ income and career concerns during the COVID-19 pandemic differ between Finland, Sweden, and the United Kingdom?

RQ2a: To what extent does current financial situation, future financial situation, childhood financial situation, generalized mistrust and loneliness relate to emerging adults’ income and career concerns, after controlling for the highest education, parental education, age, gender, and country?

RQ2b: To what extent do the associations described in RQ2a vary across countries (Finland, Sweden, and the United Kingdom)?

## Method

### Participants and Procedure

Data used in this research were from a cross-national survey conducted in April 2021 which included participants ranging in age from 18 to 75 from Finland (*n* = 1000), Sweden (*n* = 1000), and the United Kingdom (*n* = 1000). The online survey was anonymous and collected via a research company. The research company used a random sample in each country, with volunteer web panelists who willingly respond to surveys within their own interests. Panelists were reached out to in random order and were awarded with compensation and prizes for their efforts by the research company.

For the purposes of this study, the age range was limited to the ages between 18 and 34 (*n* = 976 total). The country representation of this restriction was now Finland (*n* = 309), Sweden (*n* = 324) and the United Kingdom (*n* = 343). The average age was 26.33 (*SD* = 4.60*)* and the representation of gender was 60.5% of participants identifying as female and 39.5% identifying as male.

### Measures

#### Income and Career Concerns

Income and career concerns were measured with the following questions (Hira & Mugenda, 2000; Ranta et al., 2020): *“How worried are you about the impacts of COVID-19”,* with the items being *“on your own income level“* and *“on your own career or studies”* Respondents were able to evaluate their perceptions on a scale of 1–5 (1 = *not worried at all*, 5 = *extremely worried*) (*M* = 3.13; *SD* = 1.31) (*M* = 3.07; *SD* = 1.32)*.* For the sake of clarity, reports on career or study concerns are referred to as career concerns.

#### Current Financial Situation

The current financial situation of the respondents was investigated by using the question *“How would you evaluate your financial situation at the moment?”* (Hira & Mugenda, 2000; Ranta et al., 2020)*.* Respondents were able to answer on a scale of 1–5 (1 = *extremely poor,* 5 = *extremely good*) (*M* = 3.08; *SD* = 0.92)

#### Future Financial Situation

How respondents believed their personal financial situation will develop was measured using the question *“How do you believe that your own economy will develop in the following years?”* (Hira & Mugenda, 2000; Ranta et al., 2020). Respondents could answer on a scale of 1–5 (1 = *very poorly*, 5 = *very well*) (*M* = 3.31; *SD* = 0.97)

#### Childhood Financial Situation

The childhood financial situation of the respondents was measured with the question *“Evaluate the financial sustainability of your childhood home. In the event that you had multiple homes growing up, evaluate the household which you consider most meaningful”.* Respondents could answer on a scale of 1–5 (1 = *disadvantaged*, 3 = *normal [i.e., middle class]*, 5 = *wealthy*) (*M* = 2.89; *SD* = 0.90)*.*

#### Generalized Mistrust

Generalized trust was measured by utilizing a question commonly used in surveys, such as by the World Values Survey Institute (World Values Survey WVS, 2022) and the American National Election Studies (American National Election Studies ANES, 2022), *“Generally speaking, would you say that most people can be trusted or that you can’t be too careful when dealing with people?”.* The scales used with this question vary (Lundmark et al., 2016), however for the purpose of this study, an 11-point scale was used (0 = *most people can be trusted*, 10 = *you can’t be too careful*) (*M* = 5.28; *SD* = 2.68). Due to the response scale, generalized trust will be discussed as generalized mistrust.

#### Loneliness

Loneliness was measured with a three-item loneliness scale, which was developed for this particular survey, but is comparable with full loneliness measures used in previous research (Hughes et al., 2004). The three-item question of this survey was *“Thinking of the past year, how often have you felt”,* with the follow-up questions being *“*1) *that you lack companionship?”, “*2) *left out”* and *“*3) *isolated from others”.* Respondents could answer using a scale from 1 to 3 (1 = *hardly ever*, 2 = *sometimes*, and 3 = *often*) (*M* = 2.05; *SD* = 0.55)*.* Cronbach’s alpha was .825.

#### Sociodemographic Variables

The sociodemographic variables used in our analysis include *highest education, parental education, age,* and *gender.* For highest education, participants were given five answer options 1) *Primary school or part of it,* 2) *Vocational degree (including apprenticeship,* 3) *College/high school degree,* 4) *Undergraduate degree (bachelor’s degree)*, and 5) *Master’s or Doctor’s degree* (*M* = 3.21; *SD* = 1.06)*.* For parental education, participants were asked to rate the degree level of their highest educated parent/guardian, with the options being 1) *Primary school or part of it,* 2) *Vocational degree,* 3) *College/high school degree,* 4) *Undergraduate degree,* 5) *Master’s or Doctor’s degree* (*M* = 3.35; *SD* = 1.14). Age was asked from participants in years via a drop-down list (*M* = 26.33; *SD* = 4.60). Gender was asked from participants with the options *1) Male* (39.5%), *2) Female* (60.5%)*,* and *3) Other/do not want to specify.* No respondents chose the third option.

## Results

We used IBM SPSS 26 statistical software in our analysis. As preliminary analyses, descriptive statistics of all study variables are shown in Table 1. Correlations between all study variables for the overall sample are presented in Table 2. Whereas Table 1 describes the overall sample, Table 3 presents the means and standard deviations for each country. To answer RQ1, the country scores on all study variables were compared using ANOVA (analysis of variance). For RQ2a, to predict income and career concerns for all three countries pooled together, ANCOVA (analysis of co-variance) using Univariate General Linear Models was performed. We predicted income and career concerns by self-perceived financial situation (current, future, and childhood), generalized mistrust, loneliness as well as sociodemographic variables (highest education, parental education, age, gender), controlling for the country effect. To answer RQ2b, similarly to RQ2a, both income and career concerns were predicted using ANCOVA, but this time for each country separately. ANCOVA was chosen as a study method because for RQ2a both continuous and categorical (country) variables were used. ANCOVA was also used for RQ2b to maintain consistency for the methods used and the results displayed.

**Table 1.** Descriptives of All Study Variables.

	*n* (%)	*M*	*SD*	Range		Skewness
				Potential	Actual	
Income concerns	971	3.13	1.31	1–5	1–5	−0.14
Career concerns	965	3.07	1.32	1–5	1–5	−0.12
Current financial situation	974	3.08	0.92	1–5	1–5	−0.15
Future financial situation	971	3.31	0.97	1–5	1–5	−0.30
Childhood financial situation	972	2.89	0.90	1–5	1–5	−0.20
Generalized mistrust	959	5.28	2.68	0–10	0–10	−0.11
Loneliness	975	2.05	0.55	1–3	1–3	−0.08
Highest education	973	3.21	1.06	1–5	1–5	−0.28
Parental education	908	3.35	1.14	1–5	1–5	−0.23
Age 18–34	976	26.33	4.60	18–34	18–34	−0.10
**Gender**	970	1.60	0.48	1–2	1–2	−0.43
Male	383 (39.5%)					
Female	587 (60.5%)					
**Country**	976	2.03	0.81	1–3	1–3	−0.06
Finland	309 (31.7%)					
Sweden	324 (33.2%)					
United Kingdom	343 (35.1%)

**Table 2.** Correlations Between All Study Variables.

		1	2	3	4	5	6	7	8	9	10
1	Income concerns										
2	Career concerns	.581**									
3	Current financial situation	−.175**	−.060								
4	Future financial situation	.151**	−.015	.279**							
5	Childhood financial situation	.002	−.040	.249**	.198**						
6	Generalized mistrust	.121**	.128**	−.017	−.003	.049					
7	Loneliness	.205**	−.227**	−.108*	−.054	−.051	.092**				
8	Highest education	−.054	.022	.161**	.054	.107**	−.114**	−.050			
9	Parental education	−.032	.052	−.147**	.088**	−.182**	−.034	.021	.421**		
10	Age 18–34	−.017	−.106**	.030	−.031	.041	−.014	−.074*	.183**	−.016	
11	Gender (1 male, 2 female)	.018	−.041	−.065*	−.040	−.036	.046	.073*	.092**	−.007	−.033

*Note.* *p < .05, **p < .01.

### Income and Career Concerns in Finland, Sweden, and the United Kingdom

To answer the first research question (RQ1), we used ANOVA to compare the prevalence of income and career concerns in the three countries (Finland, Sweden, and the United Kingdom) (Table 3). The results showed that income concerns (ranked between 1–5) were the highest in the United Kingdom (*M* = 3.38, *SD =* 1.26), followed by Sweden (*M* = 3.03, *SD* = 1.29) and Finland (*M* = 2.96, *SD* = 1.35). Bonferroni post-hoc test detected significant differences between the United Kingdom and Finland (Δ*M =* .466, *p <* .001), as well as the United Kingdom and Sweden (Δ*M =* .412, *p <* .001). Similarly, career concerns were the highest in the United Kingdom (*M* = 3.35, *SD* = 1.32), followed by Sweden (*M* = 2.94, *SD* = 1.37) and Finland (*M* = 2.89, *SD* = 2.89). Bonferroni post-hoc test revealed significant differences between the United Kingdom and Finland (Δ*M =* .419, *p <* .001) and the United Kingdom and Sweden (Δ*M =* .349, *p <* .001). In sum, the results suggest that individuals from the United Kingdom were more likely to experience income and career concerns than in Finland and Sweden. For gender, which was a dichotomous variable, we used the chi-square test, which showed that there is no relation between gender and country (χ^2^ [4] = 2.067, *p* = .723). In the whole sample, women scored higher than men in income concerns (Δ*M* = .219; *t*[2972] = 4.457, *p* < .001) and career concerns (Δ*M* = .209; *t*[2966] = 4.165, *p* < .001).

**Table 3.** Multiple Comparisons in Finland, Sweden, and the United Kingdom.

	*Finland* (*n* = 283)		*Sweden* (*n* = 285)		*United Kingdom* (*n* = 307)		*F*	*p*
	*M*	*SD*	*M*	*SD*	*M*	*SD*		
Income concerns	2.96^a^	1.35	3.03^b^	1.29	3.38^ab^	1.26	9.832	**<.001**
Career concerns	2.89^a^	1.31	2.94^b^	1.37	3.35^ab^	1.32	12.571	**<.001**
Current financial situation	2.96^a^	.937	3.09	.866	3.17^a^	.962	4.169	**.016**
Future financial situation	3.39	.928	3.30	.995	3.24	.987	1.786	.168
Childhood financial situation	2.82	.894	2.89	.856	2.95	.964	1.563	.210
Generalized mistrust	5.15	2.78	5.30	2.46	5.38	2.80	.603	.547
Loneliness	2.03	.600	2.01^a^	.550	2.12^a^	.517	4.03	**.018**
Highest education	2.98^ab^	1.18	3.20^ac^	1.00	3.43^bc^	.964	14.96	**<.001**
Parental education	3.19^a^	1.30	3.31	1.04	3.52^a^	1.07	6.31	**.002**
Age	26.3	4.62	26.2	4.72	26.7	4.48	.026	.975

*Note.* Bonferroni post-hoc test was used. Mean scores that share the same superscript are statistically significantly different.

**In bold**—significant results at *p* < .05 level.

### Factors Associated with Income and Career Concerns

To answer the second research question (RQ2a), we carried out two separate ANCOVAs to predict income concerns and career concerns (Table 4). First, the results showed that income concerns were negatively predicted by current financial situation (*B* = −.196*, p* < .001*)*, future financial situation (*B* = −.170*, p* < .001*)*, and positively predicted by childhood financial situation (*B* = .103*, p =* .040)*.* This suggests that the more positive one is about their own current financial situation and the more positively one views their financial situation in the future, the less one is concerned about income. In contrast, the more positively one refers to their financial situation in childhood, the more income concerns one reports. Higher loneliness also predicted higher income concerns (*B* = .413*, p* < .001). We also found that generalized mistrust and sociodemographic variables (highest education, parental education, age, and gender) were not related to income concerns. However, income concerns were predicted by one’s country, supporting ANOVA results that United Kingdom scored higher than Finland and Sweden. Effect sizes in the ANCOVA analyses (Table 4) are measured by partial eta squared (η2) (Kahn, 2017). A result of 0.01 indicates a small effect, 0.06 indicates a medium effect, and 0.14 indicates a large effect. Out of the significant predictors, current financial situation (0.018) and future financial situation (0.015) reported small effect sizes. Childhood financial situation had a very small effect size (0.005) and loneliness a small effect size, albeit slightly higher than small (0.032). For the country predictor the effect size was small (0.023).

**Table 4.** Univariate General Linear Model Predicting Income and Career Concerns.

	Dependent Variables									
	Income Concerns (Adjusted *R*^*2*^ = .103)					Career Concerns (Adjusted *R*^*2*^ = .086)				
Independent variables	*B*	*df*	*F*	*p*	*partial* η^2^	*B*	*df*	*F*	*p*	*partial* η^2^
Current financial situation	−.196	1	15.462	**.000**	.018	−.095	1	3.543	.060	.004
Future financial situation	−.170	1	13.142	**.000**	.015	−.016	1	.118	.731	<.001
Childhood financial situation	.103	1	4.242	**.040**	.005	.063	1	1.545	.214	.002
Generalized mistrust	.027	1	2.876	.090	.003	.037	1	5.207	**.023**	.006
Loneliness	.413	1	28.763	**.000**	.032	.459	1	34.443	**.000**	.039
Highest education	−.026	1	.307	.579	<.001	.040	1	.692	.406	.001
Parental education	.027	1	.417	.519	<.001	.017	1	.159	.691	<.001
Age	−.004	1	.197	.657	<.001	−.033	1	11.629	**.001**	.013
Gender (1 male, 2 female)	−.085	1	.927	.336	<.001	−.014	1	.025	.876	<.001
Country		2	10.223	**.000**	.023		2	8.709	**.000**	.020
Finland	−.450			**.000**	.020	−.411			**.000**	.017
Sweden	−.362			**.001**	.014	−.359			**.001**	.013
United Kingdom	0^a^					0^a^				
Error		859					854			

*Note.* In **bold** – significant results at *p* < .05.

^a^parameter set to zero because it is redundant.

Second, career concerns were predicted by generalized mistrust (*B* = .037*, p =* .023) and loneliness (*B* = .459*, p* < .001). This suggests that less trusting and lonelier emerging adults tend to express stronger concerns about their career. Interestingly, none of the self-perceived financial situation factors (current, future or childhood) predicted career concerns. Out of sociodemographic factors, only age emerged as a significant predictor (*B* = −.033, *p =* .001), suggesting that younger individuals were more concerned about their career. Finally, the results concerning countries supported the same conclusions as previously reported ANOVA results. The effect sizes for significant career concern predictors showed that generalized mistrust (0.006) had a very small effect size. Loneliness had a closer to medium effect size (0.039). Age showed an effect size of small (0.013).

### Country-Specific Predictors of Income and Career Concerns

To answer the third research question (RQ2b), ANCOVAs within each country were run to find predictors of income (Table 5) and career concerns (Table 6). In Finland, the most significant predictors of income concerns were one’s current financial situation (*B =* −.180, *p* = .046), future financial situation (*B* = −.254, *p* = .004), and loneliness (*B =* .453*, p* < .001). More negative perceptions of the current and future financial situations and higher loneliness predicted income concerns. The effect sizes for the predictors showed that one’s current financial situation (0.015) and future financial situation (0.030) had a small effect size. Loneliness was closer to medium in terms of effect size (0.042). In Sweden, one’s future financial situation (*B* = −.173, *p* = .035) and loneliness (*B* = .375*, p* = .006) were statistically significant in predicting income concerns. Effect sizes for one’s future financial situation (0.016) and loneliness (0.027) were small. Similarly to Finland, negative future financial situations and higher reported loneliness predicted higher income concerns. In the United Kingdom, current financial situation (*B =* −.255, *p* = .005), childhood financial situation (*B =* .178*, p =* .027*)* and loneliness (*B* = .361, *p* = .009) were the significant predictors for income concerns (Table 5). This suggests that weaker current financial situation, positive childhood financial situation and higher loneliness predicted higher income concerns. The effect sizes for the predictors showed that current financial situation (0.026), childhood financial situation (0.016) and loneliness (0.023) had small effect sizes.

**Table 5.** Univariate General Linear Model Predicting Income Concerns in Finland, Sweden, and the United Kingdom.

	Income concerns														
	Finland (adjusted *R*^*2*^ = .123)					Sweden (adjusted *R*^*2*^ = .057)					United Kingdom (adjusted R2 = .086)				
Independent variables	*B*	*df*	*F*	*p*	*partial* η^2^	*B*	*df*	*F*	*p*	*partial* η^2^	*B*	*df*	*F*	*p*	*partial* η^2^
Current financial situation	−.180	1	4.028	**.046**	.015	−.173	1	3.393	.067	.012	−.225	1	7.915	**.005**	.026
Future financial situation	−.254	1	8.457	**.004**	.030	−.173	1	4.467	**.035**	.016	−.086	1	1.229	.268	.004
Childhood financial situation	.108	1	1.501	.222	.005	−.026	1	.073	.787	.787	.178	1	4.914	**.027**	.016
Generalized mistrust	.018	1	.430	.513	.002	.042	1	1.771	.184	.184	.013	1	.270	.604	.001
Loneliness	.453	1	11.881	**.001**	.042	.375	1	11.994	**.006**	.027	.361	1	6.816	.**009**	.023
Highest education	−.062	1	2.226	.137	.008	.080	1	.678	.411	.002	.000	1	.000	.999	<.000
Parental education	−.032	1	.253	.616	<.001	−.017	1	.036	.850	<.001	−.059	1	.571	.451	.002
Age	−.005	1	.089	.766	.001	.017	1	1.061	.304	.004	−.025	1	2.529	.113	.008
Gender (1 male, 2 female)	−.122	1	.627	.429	.002	.001	1	.000	.997	<.001	−.143	1	.880	.349	.003
Error		272					273					296			

*Note.* In **bold** – significant results at *p* < .05.

**Table 6.** Univariate General Linear Model Predicting Career Concerns in Finland, Sweden, and the United Kingdom.

	Career concerns														
	Finland (Adjusted *R*^*2*^ = .095)					Sweden (Adjusted *R*^*2*^ = .074)					United Kingdom (Adjusted R^2^ = .086)				
Independent variables	*B*	*df*	*F*	*p*	*partial* η^2^	*B*	*df*	*F*	*p*	*partial* η^2^	*B*	*df*	*F*	*p*	*partial* η^2^
Current financial situation	−.059	1	.436	.509	.002	−.221	1	4.950	**.027**	.018	−.022	1	.077	.781	.000
Future financial situation	−.205	1	5.543	**.019**	.020	.072	1	.675	.412	.002	.083	1	1.210	.272	.004
Childhood financial situation	.087	1	.994	.320	.004	.063	1	.385	.536	.001	.029	1	.138	.710	.000
Generalized mistrust	.025	1	.790	.375	.003	.091	1	7.274	**.007**	.026	.007	1	.075	.785	.000
Loneliness	.424	1	10.621	**.001**	.038	.528	1	13.198	**.000**	.047	.389	1	8.266	**.004**	.027
Highest education	−.052	1	.744	.389	.003	.181	1	3.026	.083	.011	.088	1	.976	.324	.003
Parental education	.044	1	.488	.486	.002	−.072	1	.583	.446	.002	.015	1	.040	.841	.000
Age	−.042	1	5.755	**.017**	.021	−.019	1	.003	.955	.004	−.037	1	5.774	**.017**	.019
Gender (1 male, 2 female)	−.025	1	.026	.872	.000	−.009	1	.003	.955	.000	−.033	1	.048	.826	.000
Error		272					270					294			

*Note.* In **bold** – significant results at *p* < .05.

Second, for career concerns, the statistically significant predictors in Finland were one’s future financial situation (*B* = −.205*, p* = .019), loneliness (*B* = .424*, p* = .001), and age (*B* = −.042*, p* = .017). Weak evaluations of one’s future financial situation and higher reports of loneliness predicted experienced career concerns. Also, younger individuals were more concerned about their career and studies. Effect sizes for the predictors indicated one’s future financial situation (0.020) and age being small (0.021), with loneliness being small but closer to medium (0.038). In Sweden, the most significant predictors were one’s current financial situation (*B* = −.221, *p* = .027), generalized mistrust (*B =* .091, *p* = .007) and loneliness (*B* = .528*, p* < .001). Poor evaluations of one’s future financial situation and higher rates of distrust and loneliness predicted career concerns. The effect sizes of the predictors showed that one’s current financial situation (0.018) and generalized mistrust (0.026) had a small effect size and loneliness closer to medium (0.047). In the United Kingdom, loneliness (*B* = .389, *p* = .004) and age (*B* = −.008, *p* = .017*)* were statistically significant in predicting career concerns amongst emerging adults, meaning experienced loneliness and younger age were linked to increased career concerns (Table 6). Effect sizes for the predictors indicated that loneliness (0.027) and age (0.019) both had a small effect size.

## Discussion

This study examined the prevalence of income and career concerns of emerging adults during COVID-19 in Finland, Sweden, and the United Kingdom, as well as relating factors for said concerns. The key findings of our study particularly highlight the role of loneliness in income and career concerns in all three countries. Our results have shown us that loneliness experienced during COVID-19 was associated with both increased income and career concerns among emerging adults during the pandemic. The results also showed that emerging adults from the United Kingdom were more likely to experience both income and career concerns. On a general level, not accounting for country-specific differences, these data showed that emerging adults were more likely to experience increased income concerns if they felt that their current financial situation and future financial situation was bleak. Those who were less trusting and felt lonelier during the pandemic were more likely to experience increased career concerns. The findings on the impact of loneliness on experiencing income and career concerns support the findings of van den Berg et al. (2021), which found that loneliness increased distress and anxieties. As previous literature has shown, satisfying social connections are important when receiving support and facing economic disturbances and can add one’s ability to withstand sudden threats to personal economic sustainability, especially during the developmental stage of emerging adulthood (Ranta et al., 2020). In addition, establishing lasting romantic relationships typically takes place during emerging adulthood (Ranta, 2015), therefore therein lies the link between loneliness and potential difficulties finding a partner during the pandemic, as well as those not yet in cohabitating or serious romantic relationships being more prone to severe loneliness during the pandemic. Studies have shown that economic uncertainty increased greatly in the wake of the pandemic, which gives strength to our findings in terms of how the pandemic has, specifically, affected the increase in income and career concerns (Altig et al., 2020). Furthermore, the questionnaire items explicitly asking participants of the pandemic related effects on the items measured clarifies that these findings are indeed pandemic specific. The history of the emerging adults is unknown, however, and background factors relating to their life before COVID-19 remain a matter of conjecture in terms of how much the pandemic truly affected these emerging adults.

When looking at country-specific differences, as per our first research question, one can speculate how country-specific factors relating to societal changes experienced during the pandemic and socioeconomic structures in society play a role in the concerns emerging adults feel towards their incomes and careers. Emerging adulthood is by default a developmental stage with many uncertainties (Arnett, 2014), with financial and career development being no exception. Therefore, the pandemic and its unpredictability have the potential to amplify said uncertainty and set raised demand on social and societal support. For example, the availability of universal safety nets provided by both the Finnish and Swedish welfare systems may lessen the effects of sudden changes in one’s financial or occupational status in the face of sudden global and national economic uncertainty (Blossfield et al., 2005; Greve et al., 2021). With the United Kingdom representing a liberal regime, with less emphasis on public welfare provision, the sudden unemployment or decrease in income may be reasons for emerging adults to experience more income and career uncertainties in the United Kingdom than in Finland and Sweden (Esping-Andersen, 1990; Meyer et al., 2009). Moreover, the differences in what living in poverty is like in each country may be a contributing factor. However, since all of these three countries score relatively high on the quality-of-life index (Numbeo, 2019), the findings are particularly interesting in how more nuanced structural factors in a society can impact the development of emerging adults during societal crises. Naturally individual differences would also play a role in the experienced concerns, as well as household type (i.e., if there are dependents the participants must provide for). Furthermore, the individual use of state offered benefits remains unclear. The results of this study contribute to discussions on social policy implications, as to how different welfare regimes can improve upon their support in emerging adulthood resilience against crisis situations, particularly in order to avoid compromised life course development trajectories characteristic for the emerging adulthood phase. Future research could delve deeper into how emerging adults themselves perceive and value the welfare structures they live in and whether they deem them beneficial when coping with sudden global events such as the COVID-19 pandemic or take a more in-depth look into how emerging adults utilize, trust, or rely on social welfare systems or other support such as family and friends in the face of society wide crises.

The second research question asked what factors were related to income and career concerns in all three countries. The results indicate that weak financial situations led to increased income concerns during the COVID-19 pandemic consistently in all countries, meaning that those who have a self-perceived weaker financial situation would be more likely to be concerned about the pandemic’s effect on their income. Less optimistic evaluations of how one’s future financial situation will develop were also associated with increased income concerns. As Settersten et al. (2020) have stated, the pandemic and its effect on stable employment has implications for fulfilling emerging adulthood developmental tasks and the development of income and career trajectories.

The final research question focused on country-specific differences in the associations of financial and career concerns. Finnish emerging adults were more likely to experience income concerns if they perceived that their current financial situation and future financial situations were weak, and if they had experienced higher rates of loneliness during the pandemic. For Swedish emerging adults, these factors were weaker future financial situations and loneliness. Emerging adults from the United Kingdom were more likely to experience income concerns when current financial situations were weaker, if childhood financial situation was more lucrative, and if they had experienced loneliness during the pandemic. One could speculate whether better experienced wealth at childhood would entail higher income concerns later in life or more drastic changes in lifestyle in the face of precarity. Another aspect may be the expectations one has when the childhood home is from a wealthier background. Career concerns in Finland were more likely if one’s future financial situation was weaker and if emerging adults had experienced higher levels of loneliness and were younger in age. Emerging adults in Sweden were more likely to experience career concerns if they perceived their current financial situation as weaker, had higher levels of mistrust and experienced loneliness. For emerging adults in the United Kingdom, career concerns were associated with loneliness and younger age. These country-specific findings indicate the potential for growth in inequalities among emerging adults, which may be consequential for overall future trends in inequalities when some have been less affected by the damaging effects of the pandemic.

The different pandemic strategies in these three countries are also worth considering, as the different measures taken also lead to different disruptions in the lives of people. For example, stricter COVID-19 measures impact individuals working in certain industries more than if measures were more lenient. Impacted industries and increases in furlough or precarity in certain jobs may be a driving factor in increased income concerns. In Finland and Sweden, the likelihood of experiencing income concerns was not as high, which may be explained by the more lenient pandemic measures and stronger social support systems in place in the event of loss of employment. However, it is worth considering that the extent to which the participants in this study have accessed said benefits remain unknown. Therefore, the element of social security availability must be discussed with precaution when interpreting these results.

With generalized mistrust being somewhat impactful in terms of career concerns, and particularly in Sweden, one could postulate upon the possibility that weakened trust in others leads to less optimism regarding the social support available, therefore leading to heightened feelings of income and career concerns during the COVID-19 crisis. With higher generalized mistrust being attributed to higher risk perception, individuals with more generalized mistrust may be more inclined to experience concerns regarding their career trajectories due to the amplified perception of risk (Ahorsu et al., 2022). Furthermore, with generalized mistrust being found linked to political and civic identity, something which develops typically during emerging adulthood, the pandemic adds an interesting layer to the dynamics of how generalized mistrust may influence said development (Walker & Iverson, 2016; Nannestad, 2008). As generalized mistrust was also found to be linked to loneliness, the isolating effect of the pandemic may indeed lead to compromised trust and increased skepticism towards others, particularly when support is needed. The findings also suggest why younger participants in Finland and the United Kingdom were more likely to experience career concerns. This may be due to less defined life course trajectories and financial insecurity at a younger age.

With loneliness being an impactful factor for both concerns in all countries, therein lies the possibility to expand the research further on how previous experiences in loneliness and mental health distress have contributed to the prevalence of income and career concerns amongst emerging adults. The results showing how strongly loneliness was related to income and career concerns in all three countries was one of the more substantial findings in this study. This has great implications to expand on research regarding just how impactful loneliness can be in even unexpected contexts and how general loneliness, without the presence of a pandemic, truly influences the development of emerging adults. One could also speculate that those emerging adults who were furloughed, lost work or had lessened work hours would experience increased loneliness along with the occupational stressors causing income concerns. These findings contribute to previous studies on how lack of social support and loneliness contribute to stress (van den Berg et al., 2021), as well as the link between unemployment and increased loneliness (Morrish & Medina-Lara, 2021).

### Limitations

This study has some limitations that need to be considered. As it relies on cross-sectional, self-reported data, there is the possibility for biases to be present. Therefore, the results highlight merely one moment during the entirety of the pandemic and therefore no causal interpretations can be made. Moreover, the social and economic situations during the pandemic changed rapidly in the countries examined and therefore prompt for longitudinal research. These data used also had more representation from females (61%) than males, which may have an influence on the results. However, this was controlled for in the analysis and therefore should help maintain the integrity of the results. It is also worth noting that since the survey was translated and facilitated in three different languages, differences in interpretation of survey items may have occurred. Lastly, the direction of the associations made cannot be known, i.e., whether loneliness leads to more concerns about one’s future, or vice versa.

### Implications

The findings of this study have practical implications for expanding the understanding on how different regime systems can better provide income and career security to emerging adults when faced with a global crisis that disrupts everyday life. Furthermore, how strategic response to said crises and their implications on everyday life may lead to more impactful reactions and concerns particularly amongst emerging adults. These findings suggest the importance of addressing the isolating nature of the pandemic, despite having a wide range of digital tools to carry on practical and social functions, is impactful even in terms of income and career concerns as experienced loneliness was associated with both in all three countries. Particularly the role of loneliness in increased income and career concerns in all countries adds value to previous research on loneliness especially in the working aged population, an area which researchers believe to be lacking (Luhmann & Hawkley, 2016). With remote work being common practice even after the most crucial pandemic phase (Gupta, 2022), therein lies the interest in further loneliness research with new remote working trends overall. On a practical level, the findings show that there should be targeted programs towards supporting these vulnerable transitions taking place in emerging adulthood, especially in times of crisis. This is particularly important because social and financial exclusion have been shown to go hand in hand (Fernandez-Olit et al., 2016). Given the age cohort in question therein lies the increased risk for NEET circumstances and emerging adults becoming excluded from expected or normative income and career developmental trajectories.

### Conclusions

To conclude, this study has highlighted the factors related to income and career concerns experienced by emerging adults during the COVID-19 pandemic, with loneliness being the most prevalent of all factors measured. Emerging adults from the United Kingdom were more likely to experience both income and career concerns, which may give light into how both welfare structures and the level of government intervention (i.e., lockdowns and vast closure of businesses) during the pandemic may have attributed to less security in terms of one’s finances and career development. The general factors not accounting for country-specific differences showed that if one felt negatively about their current financial situation and their future financial situation, they were more likely to experience income concerns. Career concerns were associated with mistrust in others and loneliness, both of which have been current topics in the context of the pandemic – a crisis which demanded higher trust in the government and the fellow population, as well as had highly isolating tendencies. Other measures that were found to be country-specific were more nuanced, with loneliness being the most strongly linked to both income and career concerns in all countries.

## Supplemental Material

Supplemental Material - Income and Career Concerns Among Emerging Adults From Finland, Sweden, and the United Kingdom During COVID-19 Click here for additional data file. Supplemental Material for Income and Career Concerns Among Emerging Adults From Finland, Sweden, and the United Kingdom During COVID-19 by Julia A. Nuckols, Gintautas Silinskas, Mette Ranta, and Terhi-Anna Wilska in Emerging Adulthood
