# Supplementary material for: Income and Career Concerns Among Emerging Adults From Finland, Sweden, and the United Kingdom During COVID-19
Source: Emerg Adulthood. 2023 Jan 20;11(3):721–34. doi: 10.1177/21676968231153691 (PMC9871444; doi:10.1177/21676968231153691)
Supplement: Supplemental Material - Income and Career Concerns Among Emerging Adults From Finland, Sweden, and the United Kingdom During COVID-19 [file sj-pdf-1-eax-10.1177_21676968231153691.pdf]

Emerging **Adu/flood**

Open Practices Disclosure Form and Badge Aglpioation

Manuscript Title: Income and Career Concerns Among Emerging Adults from Finland, Sweden, and the United Kingdom during COVID-19

Manuscript #: EA-22-0106.R3 **Corresponding Author: Julia Nuckols**

Articles accepted to *Emerging Adulthood* after September 1, 2018 are eligible to earn badges that recognize open scientific practices: publicly available data, material, or preregistered research plans. Please read more about the badges on the journal home page, and you can also find information on the Open Science Framework wiki.

Please check one of the following boxes:

( ] I am interested in applying for a badge and have included the necessary information below.

[X] I am not interested in applying for a badge (please sign at the end of the form and return to the production office)

To apply for one or more badges acknowledging open practices, p|ease check the box(es) corresponding to the desired badge(s) below and provide the information requested in the relevant sections. To qualify for a badge, you must provide a URL, doi, or other permanent path for accessing the specified information in a public, open-acoess repository. Qualifying public, open•access repositories are **committed to preserving** data, **materials,** and/or registered **analysis** plans and keeping them publicly acceaslble via the web in perpetuity. Examples include the Open Science Framework OFFS) and the various Dataverse networks. Hundreds of other qualifying data/materials repositories are listed at htto //re3data.org/. Preregistration of an analysis plan must take place via a publicly accessible registry system (e.g., OSF, CIinicalTnials.aov or other trial registries in the WHO Registry Network, institutional registration systems). Personal websites and most departmental **websites do** not qualify as rapositones.

Authors who wish to publicly post third-party material in their data, materials, or preregistration plan must have the proper authority or permission agreement in order to do so.

There are circumstances in which it is not possible or advisable to share any or all data, materials, or a research plan publicly. For example, there are cases in which sharing participants' data could violate confidentiality. If you would like your article to include an explanation of such circumstances and/or pro?de links to any data or mateñals you have made availabl ven if not under conditions eligible to earn a badg you should do so as part of the Transparency and Openness Statement that must be included with your article.

This form is based on a template provided by the Open Science Framework, fittps://osf.io/Sfndw/

[ ] Open Data Badge

1. Provide the URL, doi, or other permanent path for accessing the data in a public, open-access repository:

   [ ] Confirm that there is sufficient information for an independent researcher to reproduce all of the reported results, including codebook if relevant.

[ ] Open Materials Badge

1. Provide the URL, doi, or other permanent path for accessing the materials in a public, open-access repository:

   ] Confirm that there is sufficient information for an independent researcher to reproduce all of the reported methodology.

[ ] Preregistered Badge

1. Provide the URL, doi, or other permanent path to the registration in a public, open-access repository:

2. Was the analysis plan registered prior to examination of the data or observing the outcomes? Please note, *Emerging Adulthood* only issues pre-registration badges for manuscripEs that contain pre-regisfered analyses.

3. Were there additional registrations for the study other than the one reported? If yes, provide links and explain.

4. Were there any changes to the preregistered analysis plan for lhe primary confirmatory analysis? If yes, explain.

5. be alloftheanalysesdescdbedinthe egiste ed pan +eQoRed in{heafce? lfno. expan.

This form is based on a template provided by the Open Science Framework, hftps://osf.io/Sfndw/

No badge will be awarded if (1) is not provided and/or (2) is answered “no,” or if (3) is answered “yes” without strong justification, or if (5) is answered ’no” without strong justification.

By signing below, authors affirm that the above information is accurate and complete, that any third-party material has been reproduced or otherwise made available only with the permission of the original author or copyright holder, and that publicly posted data do not contain information that would allow individuals to be identified without consent.

Signature: ______________________________ Date: 10/01/2023

Name: JULIA NUCKOLS

This form is based on a template provid ed by fhe Open Science Framework, https://osf.io/5fndw/
